# International values for haemoglobin distributions in healthy pregnant women

**DOI:** 10.1016/j.eclinm.2020.100660

**Published:** 2020-12-02

**Authors:** Eric O. Ohuma, Melissa F. Young, Reynaldo Martorell, Leila Cheikh Ismail, Juan Pablo Peña-Rosas, Manorama Purwar, Maria Nieves Garcia-Casal, Michael G. Gravett, Mercedes de Onis, QingQing Wu, Maria Carvalho, Yasmin A. Jaffer, Ann Lambert, Enrico Bertino, Aris T. Papageorghiou, Fernando C. Barros, Zulfiqar A. Bhutta, Stephen H. Kennedy, Jose Villar

**Affiliations:** a Maternal, Adolescent, Reproductive and Child Health (MARCH) Centre, London School of Hygiene and Tropical Medicine (LSHTM), London, UK; b Hubert Department of Global Health, Emory University, Atlanta, Georgia; c Clinical Nutrition and Dietetics Department, University of Sharjah, Sharjah, United Arab Emirates; d Nuffield Department of Women's and Reproductive Health, University of Oxford, Oxford, UK; e Department of Nutrition and Food Safety, World Health Organization, Geneva, Switzerland; f Nagpur INTERGROWTH-21st Research Centre, Ketkar Hospital, Nagpur, India; g Departments of Obstetrics and Gynecology and of Global Health, University of Washington, Seattle, WA, USA; h Department of Ultrasound, Beijing Obstetrics and Gynecology Hospital, Capital Medical University, Beijing, China; i Department of Obstetrics and Gynaecology, Faculty of Health Sciences, Aga Khan University Hospital, Nairobi, Kenya; j Department of Family and Community Health, Ministry of Health, Muscat, Oman; k Oxford Maternal and Perinatal Health Institute, Green Templeton College, University of Oxford, Oxford, UK; l Unit of the University, AOU City of Health and Science of Turin, Turin, Italy; m Programa de Pós-Graduação em Saúde e Comportamento, Universidade Católica de Pelotas, Pelotas, Brazil; n Center for Global Child Health, Hospital for Sick Children, Toronto, Canada

**Keywords:** International Haemoglobin values, Healthy pregnant women, INTERGROWTH-21st, Anaemia

## Abstract

**Background:**

Anaemia in pregnancy is a global health problem with associated morbidity and mortality.

**Methods:**

A secondary analysis of prospective, population-based study from 2009 to 2016 to generate maternal haemoglobin normative centiles in uncomplicated pregnancies in women receiving optimal antenatal care. Pregnant women were enrolled <14 weeks’ gestation in the Fetal Growth Longitudinal Study (FGLS) of the INTERGROWTH-21^st^ Project which involved eight geographically diverse urban areas in Brazil, China, India, Italy, Kenya, Oman, United Kingdom and United States. At each 5 ± 1 weekly visit until delivery, information was collected about the pregnancy, as well as the results of blood tests taken as part of routine antenatal care that complemented the study's requirements, including haemoglobin values.

**Findings:**

A total of 3502 (81%) of 4321 women who delivered a live, singleton newborn with no visible congenital anomalies, contributed at least one haemoglobin value. Median haemoglobin concentrations ranged from 114.6 to 121.4 g/L, 94 to 103 g/L at the 3^rd^ centile, and from 135 to 141 g/L at the 97^th^ centile. The lowest values were seen between 31 and 32 weeks’ gestation, representing a mean drop of 6.8 g/L compared to 14 weeks’ gestation. The percentage variation in maternal haemoglobin within-site was 47% of the total variance compared to 13% between sites.

**Interpretation:**

We have generated International, gestational age-specific, smoothed centiles for maternal haemoglobin concentration compatible with better pregnancy outcomes, as well as adequate neonatal and early childhood morbidity, growth and development up to 2 years of age.

**Funding:**

Bill & Melinda Gates Foundation Grant number 49038.

**Research in context**

**Evidence before this study**

Anaemia in pregnancy is a global health problem with associated morbidity and mortality. No study has reported normative international centiles for maternal haemoglobin in pregnancy according to gestational age using prospectively collected data from healthy women with uncomplicated pregnancies (low-risk).

**Added value of this study**

This study is the first to provide novel data on maternal haemoglobin normative centiles using prospective, population-based data from eight geographically diverse areas following the WHO prescriptive approach. The gestational age-specific haemoglobin distributions for healthy pregnant women with uncomplicated pregnancies are useful for defining anaemia and are compatible with normal distributions of functional outcomes such as fetal growth, neonatal morbidity, and infant and child growth and development up to 2 years of age.

**Implications of all the available evidence**

The current WHO cut-off points for defining anaemia in pregnancy and its severity, are largely derived from the recommendations of WHO expert technical consultations. Our study provides evidence to help inform re-examination of WHO haemoglobin cut-off points for defining anaemia in pregnancy.

Alt-text: Unlabelled box

## 1 Introduction

Anaemia in pregnancy is a global nutritional problem associated with increased risks of maternal mortality [1], Caesarean section [2], low birth weight (LBW), small-for-gestational age, preterm birth, and perinatal and neonatal mortality [3,4]. It is estimated that, in 2016, 40% of pregnant women (95% CI: 36.4 to 44.7%) had anaemia globally, with the highest prevalence in the WHO regions of South-East Asia (58.2%) and the lowest in the Americas (25.5%) [5]. As of 2020, prevalence of anaemia in women aged 15 to 49 years, by pregnancy status (percentage) is considered as an indicator to assess progress towards Sustainable Development Goal 2: End hunger, achieve food security and improved nutrition and promote sustainable agriculture by the United Nations Statistical Commission [6] and reported in the World Health Statistics 2020 [7]. Achieving a 50% reduction in anaemia among women 15–49 years of age is relevant to the UN Sustainable Development Goal [8] and World Health Organization (WHO) global nutrition target for 2025 [9].

In 1958, a WHO Study Working Group on Iron Deficiency Anaemia met in Geneva, Switzerland and determined that, for adult pregnant women, haemoglobin (Hb) concentrations below 100 g/L were indicative of anaemia [10]. This cut-off was based on the analysis of haematological data derived from studies of apparently normal populations and was intended for use in nutritional surveys in different parts of the world. In 1967, WHO defined maternal anaemia as an Hb concentration below 110 g/L at any gestational age [11]. Other thresholds were suggested by the US Centers for Disease Control and Prevention (CDC), expert clinical organisations, and individual clinical and research laboratories [3,12]. WHO currently recommends Hb cut-offs below which individual women should be defined as anaemic, by trimester of pregnancy (first trimester: <110 g/L; second trimester: <105 g/L; third trimester: <110 g/L) [13,14]. WHO also considers a normal Hb range in women by trimester as the assessment basis for blood transfusion, when needed [15].

The US CDC cut-offs were derived from gestational month-specific 5^th^ centile values for pooled Hb data from four small European studies (UK 1982, *n* = 45; Sweden 1975, *n* = 50; Finland 1980, *n* = 32; Finland 1977, *n* = 267) involving ‘healthy’ women [16], [17], [18], [19]. The trimester-specific cut-offs were based on the mid-trimester values [20]; cut-offs for the first trimester, when most women were initially seen for antenatal care, were based on a late-trimester value.

The need for better quality data to redefine the cut-offs in both pregnancy and childhood has been recognised for some time. In 2015, WHO initiated a project to review the Hb concentration cut-offs used to define anaemia in individuals and populations; review its social, biological, behavioural, environmental, and contextual determinants, and assess the expected impact of public health interventions for preventing and controlling anaemia and Hb concentrations [21]. In 2018, experts, policymakers and programme implementers met to review key information and identify knowledge gaps relating to the diagnosis of anaemia [22]. The paucity of data relating maternal, newborn and child health outcomes to Hb values was also confirmed in a recent review, which stressed that not enough is known about the gestational age-specific Hb thresholds that predict health risk/protection for mother and infant [23].

To gather evidence to support updating recommendations for Hb concentrations during pregnancy that are associated with good maternal and child health outcomes, pooling high-quality individual-level data from prospective cohort studies was considered an imperative [23]. Thus, WHO searched for data sets that could provide the required populations, according to the characteristics identified previously for establishing international standards for human growth and development [24].

The Fetal Growth Longitudinal Study (FGLS), a major component of the INTERGROWTH-21^st^ Project [25], was considered an appropriate source for such information because: a) populations were selected within defined geographical areas with absent or low levels of major, known, non-microbiological contamination, at an altitude of less than 1600 m; in addition, high educational level and socio-economic status, and low perinatal mortality rates, were present at population level; b) the women enrolled from these populations were healthy, educated and well-nourished; c) they had received quality-of-care pregnancy and delivery health services; d) their pregnancies were well-dated; e) they had very low rates of adverse maternal, perinatal and neonatal outcomes, and f) their children had satisfactory growth and neurodevelopment at 2 years of age [25], [26], [27], [28], [29].

Hence, this paper provides, for the first time, normative Hb trajectories to establish gestational age-specific distributions that are compatible with functional health outcomes up to 2 years of age, as well as normal population thresholds for Hb in pregnancy. These parameters complement the international standards for early and late fetal growth, maternal weight gain, symphysis fundal height, and newborn size and body composition produced from the same FGLS data set [29], [30], [31], [32], [33], [34].

## 2 Methods

This work is reported following the STROBE guidelines [35].

### 2.1 Study design

A secondary analysis of prospective, population-based, longitudinal, observational cohort study from 2009 to 2016 to generate maternal haemoglobin normative centiles in uncomplicated pregnancies in women receiving optimal antenatal care. The INTERGROWTH-21^st^ Project consisted of several interrelated studies with the principal aim of evaluating growth, health, nutrition and development from less than 14 weeks’ gestation to 2 years of age, using the same conceptual framework as the WHO Multicentre Growth Reference Study (MGRS) [36].

### 2.2 Study site and population selection

#### 2.2.1 Setting

The INTERGROWTH-21^st^ Project was carried out between 2009 and 2016 across eight diverse geographically delimited urban areas in: Pelotas (Brazil), Turin (Italy), Muscat (Oman), Oxford (UK), Seattle (USA), Beijing (China), Nagpur (India), and Nairobi (Kenya) [25]. The selection criteria at the cluster level were: the areas had to be located at an altitude <1600 m above sea level with a low risk of fetal and infant growth and developmental disturbances, as well as an absence or low levels of major, known, non-microbiological contamination. Within each area, all institutions classified locally as “private” or “corporation” hospitals and/or serving the middle to upper socio-economic population were selected, provided that most institutional deliveries from the target population took place there. Women receiving antenatal care had to plan to deliver in these institutions or in a similar hospital located in the same geographical area.

#### 2.2.2 Participants

The participants were selected based upon criteria for optimal health, nutrition, education and socioeconomic status, needed to construct international standards [24]. At each study site, we recruited women with no clinically relevant obstetric, gynaecological or medical history, who initiated antenatal care in early pregnancy i.e., <14^+0^ weeks’ gestation by menstrual dates, and met the entry criteria of optimal health, nutrition, education and socio-economic status. A detailed description of the entry criteria and definitions has been published previously [25]. For example, adequate nutritional status was defined in the first trimester according to maternal height (≥153 cm), body mass index (BMI, ≥18.5 and <30 kg/m^2^), Hb level (≥110 g/L), and not receiving treatment for anaemia or following any special diets (e.g., vegetarian with no animal products). This resulted in a group of educated, affluent, clinically healthy women with adequate nutritional status, who by definition were at low risk of adverse maternal and perinatal outcomes.

The FGLS exclusion criteria included hypertension (defined as systolic ≥140 mmHg or diastolic ≥90 mmHg) in a past pregnancy or in the first trimester of the present pregnancy; chronic hypertension on treatment, and a past history of preeclampsia, eclampsia or Haemolysis Elevated Liver enzymes and Low Platelets (HELLP) syndrome. FGLS also excluded women if their pregnancies became complicated by criteria specified a *priori*, including fetal death, congenital anomaly, severe or catastrophic medical morbidity not evident at enrolment (such as cancer or HIV), severe unanticipated conditions related to the pregnancy (such as severe preeclampsia or eclampsia), and those identified during the study who no longer fulfilled the entry criteria (e.g. women who started smoking during pregnancy or had an episode of malaria) [25].

Gestational age was calculated from the date of the last menstrual period provided: the woman had a regular 24–32-day menstrual cycle, she had not been using hormonal contraception or breastfeeding in the preceding 2 months, and any discrepancy between the gestational ages based on last menstrual period and crown-rump length, measured by ultrasound between 9^+0^ and 13^+6^ weeks’ gestation, was 7 days or less. The dating scan was undertaken using standard study criteria for measuring crown-rump length [37]. Dedicated research staff then performed an ultrasound scan every 5 weeks (± 1 week) until delivery to assess fetal growth. At each visit, information was collected about the pregnancy, as well as the results of blood tests (including Hb) taken as part of routine antenatal care that was provided separately to the study's requirements. The gestational age at which those tests were taken varied depending on local protocols as this was a pragmatic study that aimed to mimic routine clinical practice in the different settings.

### 2.3 Haemoglobin analysis

The primary objective of this analysis of the FGLS data was two-fold: (a) to describe Hb ranges and trajectories in a population of optimally healthy women with good pregnancy, perinatal and neonatal outcomes, whose children had satisfactory postnatal growth and development up to 2 years of age so as to establish gestational age-specific distributions and populations thresholds for normal Hb in pregnancy, and (b) to define prevalence thresholds to diagnose adequate Hb concentrations in pregnancy at the individual level and the prevalence of anaemia at population level.

The Hb tests were taken as part of routine antenatal care, i.e., in relation to laboratory tests, 1) we relied on collecting the results of available routine blood tests; 2) the commercially available instruments for assessing Hb were not standardised across sites; and 3) information on the use of preventive or therapeutic iron and folic acid-containing supplements or calcium supplements, was collected from medical records. Although the eight study sites were not asked to follow a specific protocol, we have documented carefully the biochemical methods of Hb determination they used. All sites assessed Hb concentration from venous blood samples using commercially available methods (automatised colorimetry, automatised turbidimetry, high efficiency liquid chromatography, sysmex autoanalyser, automated flow fluorescent analyser, photometric method using automated cell counter, high-efficiency liquid chromatography and cyanide-free sodium lauryl sulphate) of Hb assessment that are widely used in routine patient care and considered highly reliable [38].

### 2.4 Statistical methodology

Our overall aim was to produce Hb centiles that change smoothly with gestational age and maximise simplicity without compromising model fit. We followed the same statistical methodology and approach previously described [39,40] for the analyses of already published international standards [29,30,33,41].

The first step was to assess the variation in maternal Hb across different study sites to determine whether we could pool the data to estimate international normative values. The criteria used to judge similarities among study sites was based on WHO recommendations for analysing human growth data [42]. In brief, we first inspected the data visually comparing patterns across sites. We then applied variance component analysis (analysis of variance (ANOVA)) to calculate the percentage of variance in the longitudinal maternal Hb concentrations from variance between sites and the estimated variance in individuals within a site (within-site variance). We treated gestational age as a fixed effect, whereas sites and individuals were treated as random effects in a multi-level linear regression model.

Having satisfied the criteria for pooling, we used the pooled data to construct smoothed centiles of maternal Hb according to gestational age using fractional polynomial regression that models the mean and standard deviation (SD) separately as a smooth function of gestational age. The best fitting powers for the mean and SD of maternal Hb according to gestational age were provided by the second and first-degree fractional polynomials, respectively. Goodness of fit of the resultant models was assessed as previously described for the INTERGROWTH-21^st^ data by Ohuma and Altman [39], i.e., visual inspection of the overall model fit by comparing empirical centiles (calculated per completed week of gestation, e.g. 38 weeks = 38^+0^ - 38^+6^ weeks’ gestation) to the fitted centiles, a plot of the residuals (observed values minus fitted values) according to gestational age, a quantile-quantile (Q‐Q) plots of the residuals to assess normality, and a plot of fitted z-scores across gestational ages.

We then conducted various sensitivity analyses: 1) comparing the fitted smoothed centiles of longitudinal Hb data (*n* = 3502 women, 9954 observations) to a cross-sectional random sample of Hb data between 14^+0^ and 40^+0^ weeks’ gestation for each woman included in the study (*n* = 3502 observations) to evaluate whether multiple Hb values per women resulted in reduced error variance and consequently reduced variance of the estimated centiles; 2) comparing the total pooled sample (*n* = 3502 women) with the sample (*n* = 3364 women) that excluded those mothers who delivered preterm, i.e. less than 37 weeks’ gestation (*n* = 138 women), and then superimposing the two sets of fitted centiles to evaluate whether there were differences in maternal Hb among women delivering preterm compared to term newborns; and 3) excluding each site's Hb data one at a time, refitting the centiles (seven sites’ data), and comparing the fitted (i.e., 3^rd^, 50^th^ and 97^th^ centiles) on the basis of fractional polynomial regression between the pooled data (eight sites) and the reduced datasets (one site excluded at a time) to establish whether there was any site-specific influence to the derived smoothed pooled maternal Hb centiles.

Descriptive analyses were used to summarise data on supplementation information that was available and collected as part of routine care. The supplementation was provided as prophylaxis as applied in routine practice following country-specific guidelines. In addition, for each site, we calculated empirical Hb centiles (specifically, 3^rd^, 5^th^, 10^th^, 50^th^, 90^th^, 95^th^ and 97^th^ centiles) and then computed the median across all eight sites to obtain Hb centiles for situations where gestational age is unknown. All analyses were performed in STATA, version 15, software (StataCorp LP, College Station, TX, USA).

The next step was to decide the approach for establishing thresholds for Hb concentration to define anaemia at individual level. Such thresholds are applied to judge the location of a single value in relation to the median of the normative distribution, i.e. to assess an individual's status.

The definition of “normality” is conventionally set at 2 SD below (or above) a standard or normative median, but this is frequently rounded up to the 3^rd^ (or 97^th^) centile in most international guidelines and literature [43]. We used this value to define the most severe threshold of Hb concentration in this population of healthy pregnant women.

### 2.5 Patient and public involvement

The INTERGROWTH-21^st^ Steering Committee included voluntary lay member representation during the design and implementation of the project [44]. We plan to involve pregnant women in the dissemination of results through publication in peer-reviewed journals, presentation at national conferences and involvement of maternity groups associated with the Nuffield Department of Women's & Reproductive Health, University of Oxford, and the WHO constituted Guideline Development Group – Anaemia: use and interpretation of Hb concentrations for assessing anaemia status in individuals and populations.

### 2.6 Ethical approval

The INTERGROWTH-21^st^ Project was approved by the Oxfordshire Research Ethics Committee “C” (reference: 08/H0606/139), the research ethics committees of the individual institutions and the regional health authorities where the project was implemented. All women provided informed written consent to participate in the study.

### 2.7 Role of the funding source

The funders of the study had no role in study design, data collection, data analysis, data interpretation, or writing of the report. The corresponding author had full access to all the data in the study and had final responsibility for the decision to submit for publication.

## 3 Results

### 3.1 Participants

**Fig. 1:**
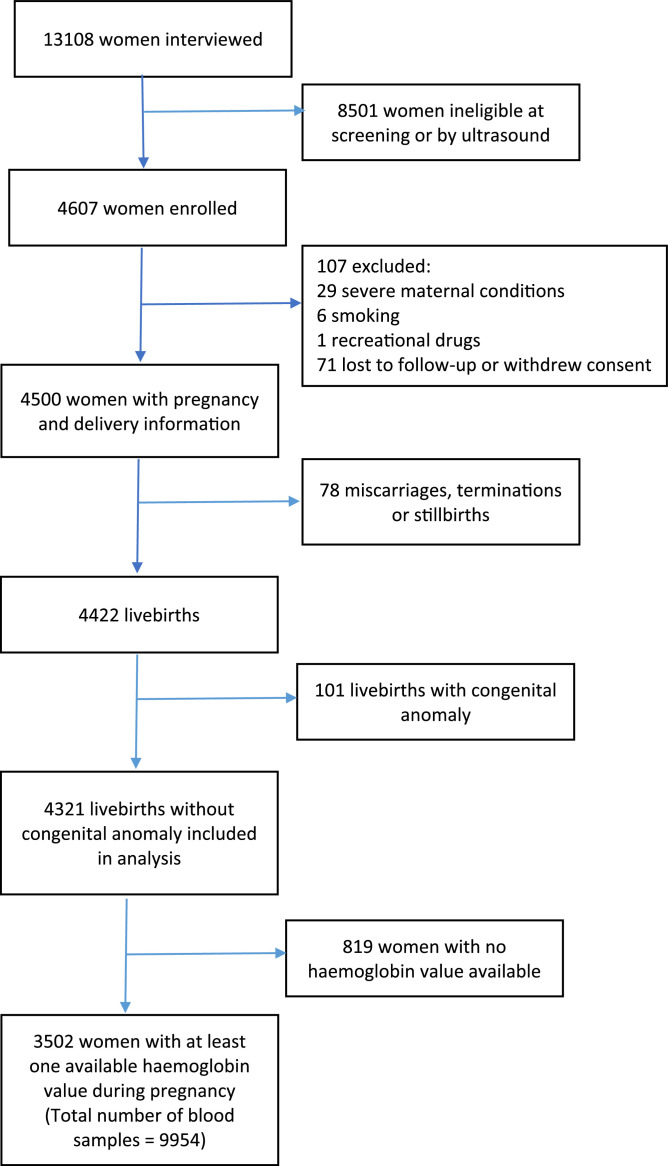
Flow diagram illustrating women enrolled in the fetal Growth Longitudinal Study.

The enrolment strategy and eligibility criteria of the INTERGROWTH-21^st^ Project, at population and individual level, have been published previously [25,29]. In brief, 13,108 pregnant women were screened at <14 weeks’ gestation. Of these, 4607 (35.1%) met the eligibility criteria, provided written informed consent and were enrolled in FGLS. The most common reasons for exclusion were maternal height <153 cm (1022/8501; 12%), BMI ≥30 kg/m^2^ (1009/8501; 12%), and age <18 or >35 years (915/8501; 11%) at screening. Seventy-one women (2%) were either lost to follow-up or withdrew consent during pregnancy. Thirty-six were excluded (29 had severe medical conditions, six took up smoking, and one used recreational drugs). A total of 4422 women delivered a liveborn singleton. Of these, 4321 (97.7%) had a baby without a congenital anomaly, they are the same cohort that contributed data to the production of the INTERGROWTH-21^st^ Fetal Growth Standards [29]. Of the 4321 women enrolled, 3502 (81.0%) contributed at least one Hb value for analysis (Fig. 1).

#### 3.1.1 Maternal, perinatal and childhood outcome data compatible with a cohort at low risk for morbidity and mortality

The socio-demographic characteristics of the complete FGLS cohort, and maternal and perinatal outcome data have been reported previously [28] and are similar to those of women included in the present analysis (Table 1). The mean maternal age was 28.0 (SD 3.8) years; 96.9% (3394/3502) of the women were married or living with a partner, and 54.5% (1907/3502) were nulliparous. Their mean BMI at enrolment (between 9 ^+^ ^0^ and 13^+6^ weeks’ gestation) was 22.9 (SD 3.0) kg/m^2^. The median gestational age at the first antenatal visit was 11.8 (SD 1.3) weeks. The preterm birth, term LBW, Caesarean section, preeclampsia and neonatal mortality rates were 3.9%, 3.0%, 24.1%, 0.7%, and 0.09%, respectively (Table 1).

**Table 1:** Baseline characteristics and maternal and perinatal outcome data for women enrolled in the Fetal Growth Longitudinal Study (FGLS) compared to those women in the present analysis.

Women	FGLS cohort(*n* = 4321)	Present analysis(*n* = 3502)
Age (years) mean (SD)	28.4 (3.9)	28.0 (3.8)
Body mass index (kg/m^2^); mean (SD)	23.3 (3.0)	22.9 (3.0)
Gestational age at first visit (weeks); mean (SD)	11.8 (1.4)	11.8 (1.3)
Years of formal education (years); mean (SD)	15.0 (2.8)	14.9 (2.9)
Married or cohabiting; n (%)	4204 (97.3)	3394 (96.9)
Nulliparous; n (%)	2955 (68.4)	1907 (54.5)
Preeclampsia; n (%)	31 (0.7)	26 (0.7)
Spontaneous onset of labour; n (%)	2868 (66.4)	2318 (66.2)
Caesarean section; n (%)	1541 (35.7)	844 (24.1)
Neonatal Intensive Care Unit admission (>1 day); n (%)	240 (5.6)	196 (5.6)
Preterm (<37 weeks’ gestation); n (%)	195 (4.5)	137 (3.9)
Term low birth weight (<2500 g; ≥37^+0^ weeks’ gestation); n (%)	128 (3.0)	104 (3.0)
Neonatal mortality; n (%)	7 (0.2)	3 (0.09)
Male; n (%)	2149 (49.7)	1742 (49.7)
Exclusive breastfeeding at discharge; n (%)	3786 (87.6)	3251 (92.8)
Birthweight (≥37^+0^ weeks’ gestation) (kg)	3.3 (0.4)	3.2 (0.4)
Birth length (≥37^+0^ weeks’ gestation) (cm)	49.4 (1.9)	49.2 (1.8)
Birth head circumference (≥37^+0^ weeks’ gestation) (cm)	33.9 (1.3)	34.0 (1.3)

In addition, the children of the women included in this analysis had low morbidity and adequate growth and development at 2 years of age [26,27] **(Supplementary Tables 1 and 2, Supplementary Fig. 1)**. For example, at 2 years of age the cohort was at the 56^th^, 55^th^ and 48^th^ centiles of the WHO Child Growth Standards for weight, length and head circumference, which helps to confirm that the original sample selected was healthy and well-nourished **(Supplementary Table 1)**. Similarly, the observed morbidity and hospitalisation rates were as expected for a sample of healthy, free-living, urban children **(Supplementary Tables 2)**. Finally, the median age at achieving the four WHO gross motor milestones matched the WHO windows of achievement for the same milestones [45] (**Supplementary Fig. 1**).

**Fig. 2:**
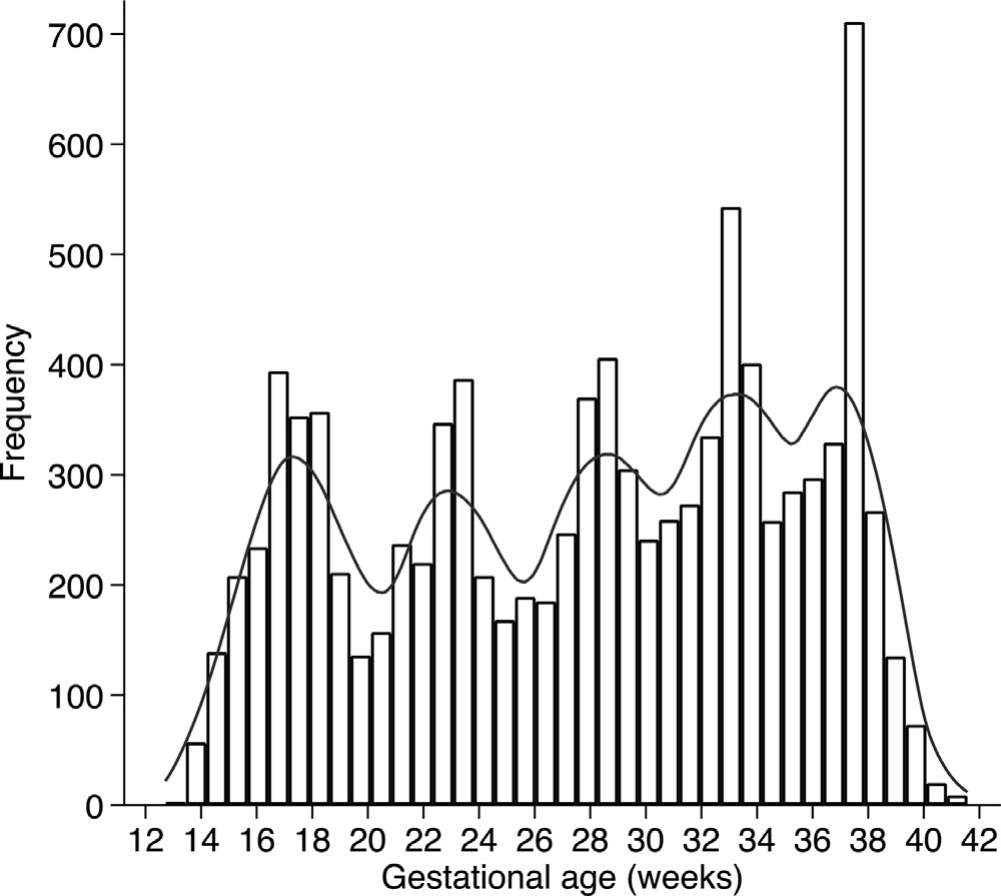
Distribution of gestational ages at which haemoglobin concentration was measured.

Maternal Hb was measured a median of twice (IQR = 1–3, range = 1–6) throughout pregnancy, resulting in 9954 values (Fig. 2). Most women (66.8%, 2338/3502) had at least two Hb measures during pregnancy; 14.6% (511/3502) had three measures, 8.1% (285/3502) four measures, and 11.1% (389/3502) five measures. Median Hb level before 15 weeks was 124 g/L in Brazil, 135 g/L in China, 116 g/L in India, 129 g/L in Italy, 129 g/L in Kenya, 117 g/L in Oman, 127 g/L in UK, and 127 g/L in USA. The contribution of each site to the study sample used in this analysis was USA 2.2% (*n* = 77 women), Kenya 8.0% (*n* = 280), Brazil 8.8% (*n* = 309), Italy 13.2% (*n* = 463), Oman 16.5% (*n* = 577), UK 16.8% (*n* = 589), India 17.1% (*n* = 600) and China 17.3% (*n* = 607).

We explored the variation in maternal Hb within and between sites and expressed it as a percentage of the total variance; the within-site variance (47.4% of the total variance) was approximately four times higher than the between-sites’ variance (12.8% of the total variance) (Table 2).

**Table 2:** Variance components analysis for fetal, newborn and childhood skeletal growth from the cohort of the INTERGROWTH-21st Project.

	fetal ultrasound measures (21)		Size at birth (21)	Infancy/childhood		During pregnancy
	1st trimester fetal CRL^a^	2^nd^ & 3^rd^ trimesters fetal HC	Newborn length^a^	Preterm infant length (33)	Infant length^b^	Present study maternal Hb
**Variance between study sites**	1.9%	2.6%	3.5%	0.2%	9.7%	12.8%
**Variance among individuals within a site**	−	18.6%	−	57.1%	60.6%	47.4%
**Residual variance**	98.1%	78.8%	96.5%	42.7%	29.7%	39.8%

a Variance among individuals for these measures could not be estimated given the cross-sectional nature of the data.

b Includes length measurements at 1 and 2 years of age. CRL: crown-rump length; HC: head circumference.

Table 2 also includes the variance component analyses similar to those previously reported for other biomarkers of growth and nutrition from early pregnancy to childhood [46]. As can be seen, the percentage of variance for these nutritional biomarkers in healthy, educated and well-nourished populations is several times higher among individuals within a site compared to between sites, with the highest figure (still only 12.8% of the total variance) for Hb in pregnancy.

**Fig. 3:**
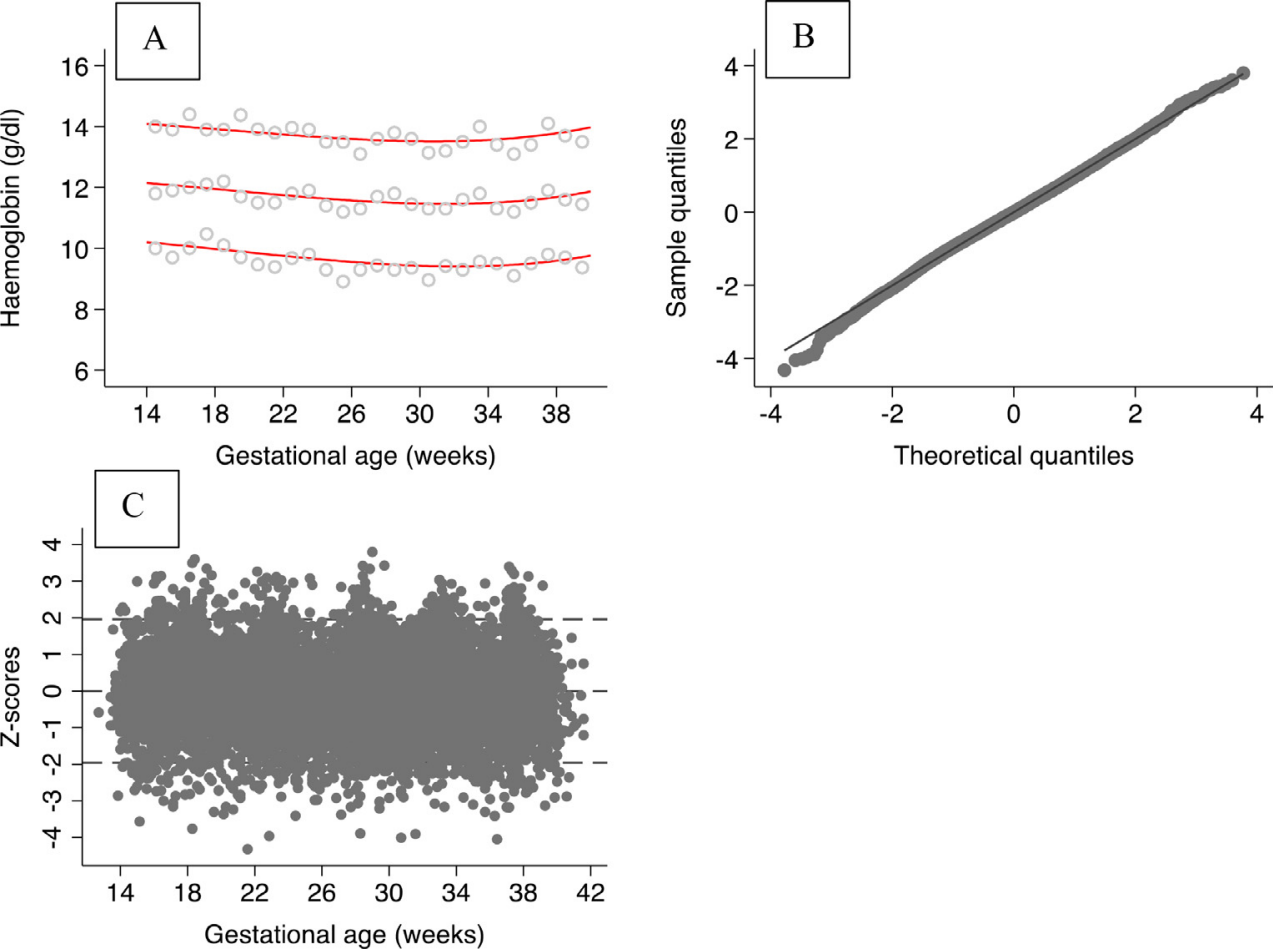
Goodness-of-fit plots showing (A) fitted 3rd, 50th and 97th smoothed centile curves of maternal haemoglobin (red solid lines) and open grey circles showing empirical values for each week of gestation (top left plot); (B) normal quantile‐quantile (Q‐Q) plots of the distribution of z‐scores (top right plot), and (C) a scatter plot of z-scores according to gestational age in weeks (bottom left plot).

Goodness of fit by gestational age-specific comparisons of empirical centiles to smoothed centile curves showed good agreement and scatter plots of z-scores by gestational age did not show any relationship with gestational age (Fig. 3).

Visual assessment of fitted smooth centiles of gestational age-specific comparisons show good agreement between the smoothed centile curves (3^rd^, 50^th^ and 97^th^ centiles) and empirical centiles. Overall, the mean differences between smoothed and observed centiles for the 3^rd^, 50^th^ and 97^th^ centiles, respectively, were small: 0.6 g/L (SD 2.5 g/L), −0.5 g/L (2.3 g/L), and −0.3 g/L (2.8 g/L) (Fig. 3, A). A normal Q‐Q plot of z‐scores (Fig. 3, B) evaluates whether the residuals have a close‐to‐normal distribution represented by a straight diagonal line cutting through the plot, in which the z-scores are normally distributed across the range of gestational ages. The distribution of z-scores according to gestational age is shown in (Fig. 3, C). Both this and the Q-Q plot, show no obvious pattern according to gestational age (constant variance).

**Fig. 4:**
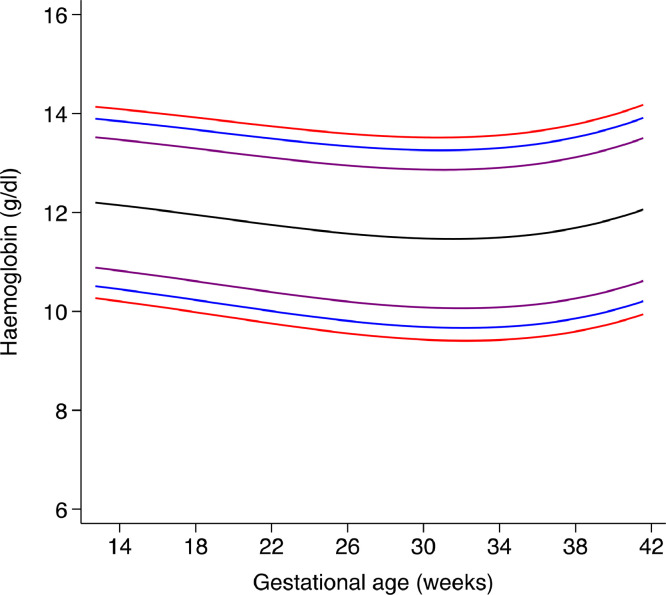
represents the smoothed, gestational age-specific, 3^rd^ (red), 5^th^ (blue), 10^th^ (purple), 50^th^ (black) 90^th^ (purple), 95^th^ (blue) and 97^th^ (red) centiles for maternal haemoglobin. (For interpretation of the references to color in this figure legend, the reader is referred to the web version of this article.)

**Fig. 5:**
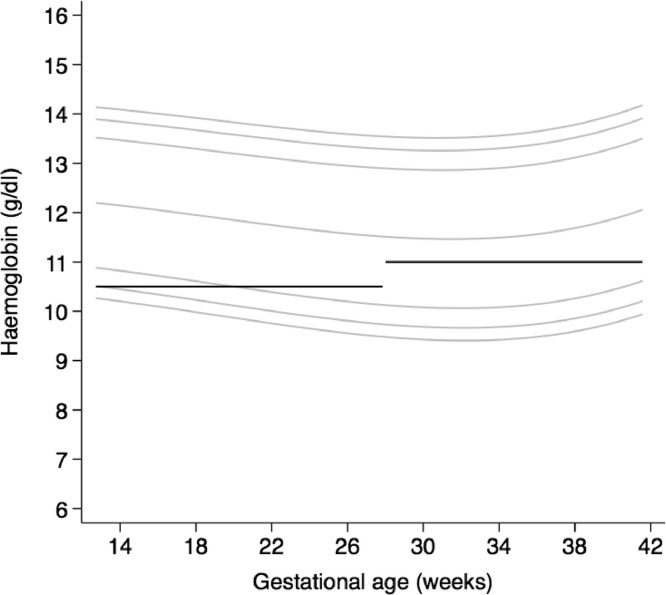
represents smoothed, gestational age-specific, 3^rd^, 5^th^, 10^th^, 50^th^, 90^th^, 95^th^ and 97^th^ centiles for maternal haemoglobin superimposed on the current recommended WHO cut-offs for pregnant women in the second and third trimesters (black solid lines).

The highest median maternal Hb concentration was at 14 weeks’ gestation (121.4 g/L) and the lowest was between 31 and 32 weeks’ gestation (114.6 g/L), but values rose progressively thereafter to a median concentration of 118.7 g/L at 40 weeks’ gestation (Table 3, Fig. 4). The current recommended WHO Hb cut-offs for pregnant women are superimposed on the smoothed, gestational age-specific centiles derived from our cohort (Fig. 5).

**Table 3:** Smoothed centiles for maternal haemoglobin (in g/L) according to exact gestational age (in weeks).

Gestational age (exact week)	3^rd^ centile	5^th^ centile	10^th^ centile	50^th^ centile	90^th^ centile	95^th^ centile	97^th^ centile
14 weeks + 0 days	102	104	108	121	135	138	141
15 weeks + 0 days	102	104	108	121	134	138	140
16 weeks + 0 days	101	103	107	121	134	138	140
17 weeks + 0 days	100	103	107	120	133	137	140
18 weeks + 0 days	100	102	106	120	133	137	139
19 weeks + 0 days	99	102	106	119	132	136	139
20 weeks + 0 days	99	101	105	118	132	136	138
21 weeks + 0 days	98	101	104	118	131	135	138
22 weeks + 0 days	98	100	104	117	131	135	137
23 weeks + 0 days	97	100	103	117	131	134	137
24 weeks + 0 days	96	99	103	117	130	134	137
25 weeks + 0 days	96	99	102	116	130	134	136
26 weeks + 0 days	96	98	102	116	129	133	136
27 weeks + 0 days	95	98	102	115	129	133	136
28 weeks + 0 days	95	97	101	115	129	133	135
29 weeks + 0 days	95	97	101	115	129	133	135
30 weeks + 0 days	94	97	101	115	129	133	135
31 weeks + 0 days	94	97	101	115	129	133	135
32 weeks + 0 days	94	97	101	115	129	133	135
33 weeks + 0 days	94	97	101	115	129	133	135
34 weeks + 0 days	94	97	101	115	129	133	136
35 weeks + 0 days	94	97	101	115	129	133	136
36 weeks + 0 days	95	97	101	116	130	134	136
37 weeks + 0 days	95	98	102	116	130	134	137
38 weeks + 0 days	96	99	103	117	131	135	138
39 weeks + 0 days	97	99	103	118	132	136	139
40 weeks + 0 days	98	100	104	119	133	137	140

**Fig. 6:**
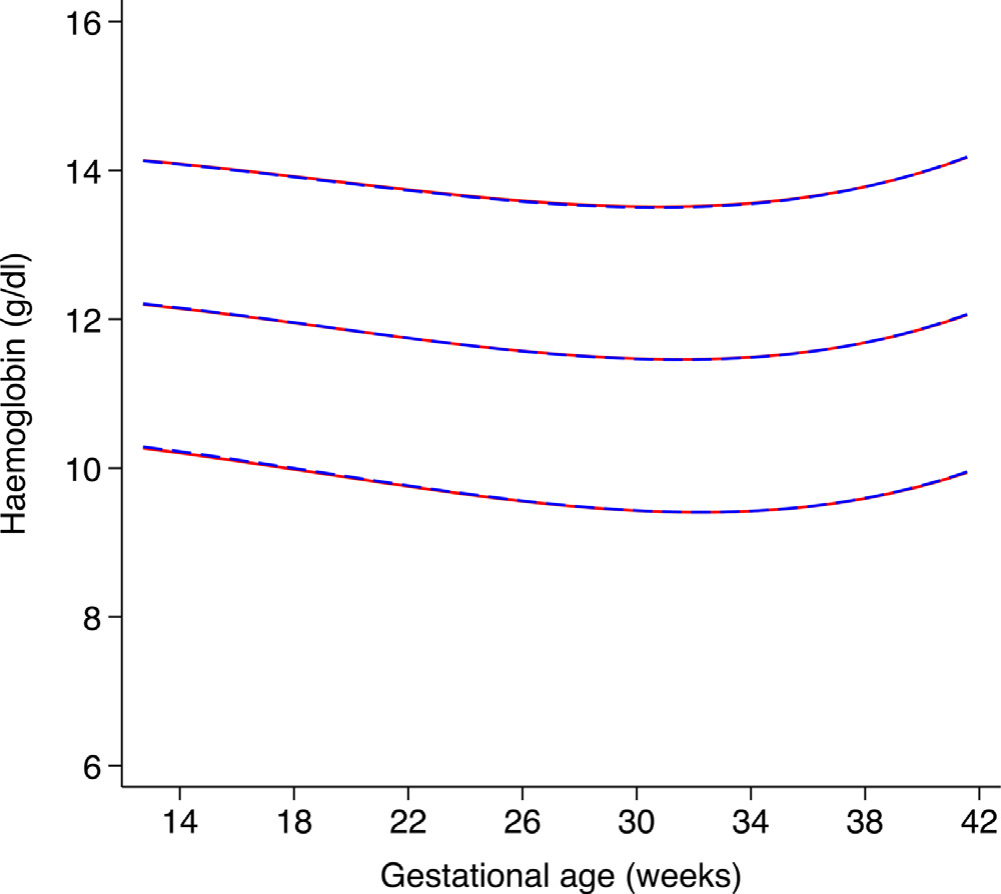
represents sensitivity analyses showing the smoothed, gestational age-specific, 3^rd^ (red), 50^th^ (red) and 97^th^ (red) centiles for maternal haemoglobin from the total Fetal Growth Longitudinal Study pooled sample (*n* = 3502) and the fitted 3^rd^ (blue), 50^th^ (blue), and 97^th^ (blue) centiles after excluding women who delivered preterm (*n* = 3364). (For interpretation of the references to color in this figure legend, the reader is referred to the web version of this article.)

**Fig. 7:**
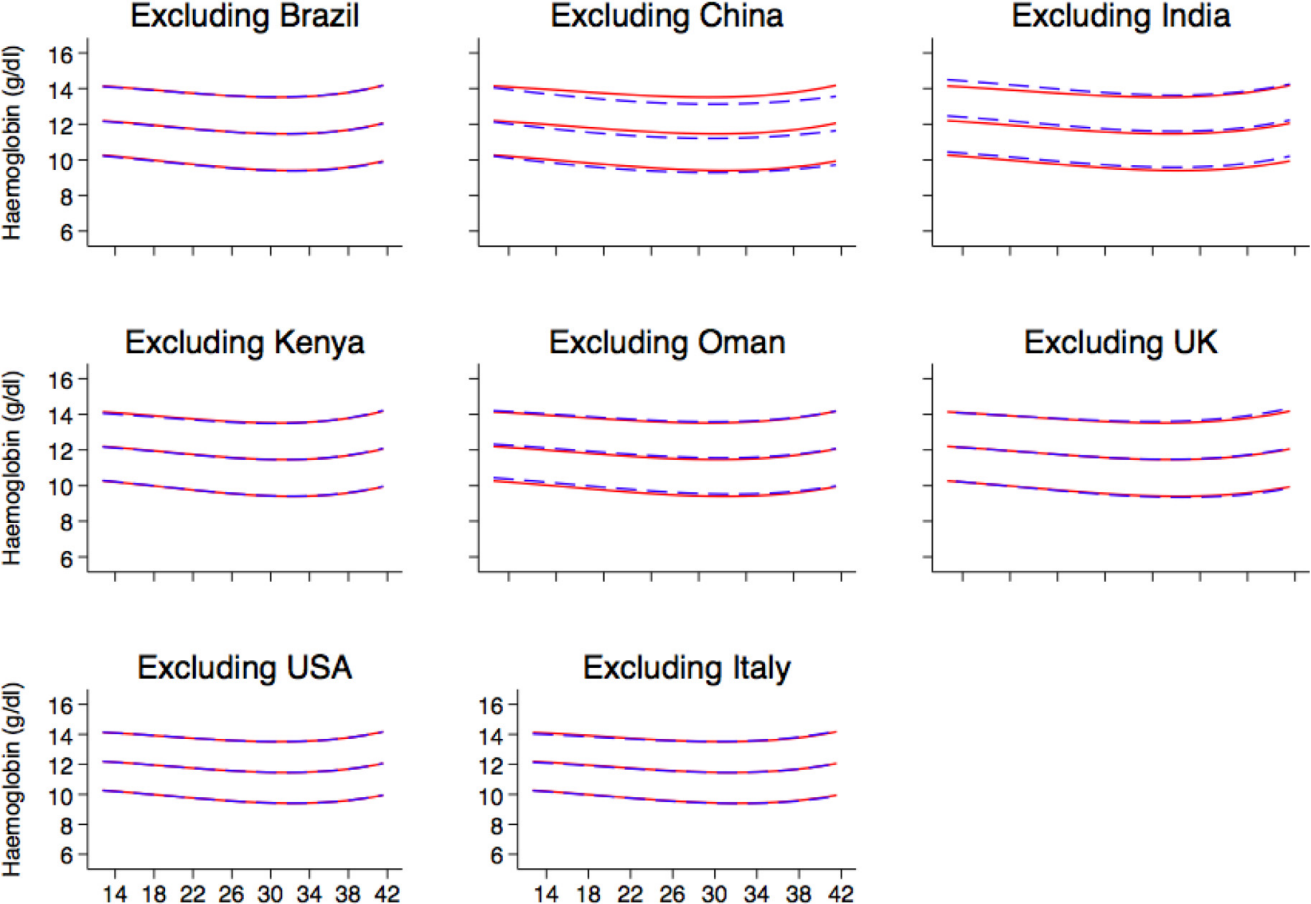
represents sensitivity analyses showing the smoothed, gestational age-specific, 3^rd^ (red), 50^th^ (red) and 97^th^ (red) centiles for maternal haemoglobin from the total Fetal Growth Longitudinal Study pooled sample (*n* = 3502) and the fitted 3^rd^(blue), 50^th^ (blue), and 97^th^ (blue) centiles after excluding maternal haemoglobin data from each country in turn. (For interpretation of the references to color in this figure legend, the reader is referred to the web version of this article.)

**Fig. 8:**
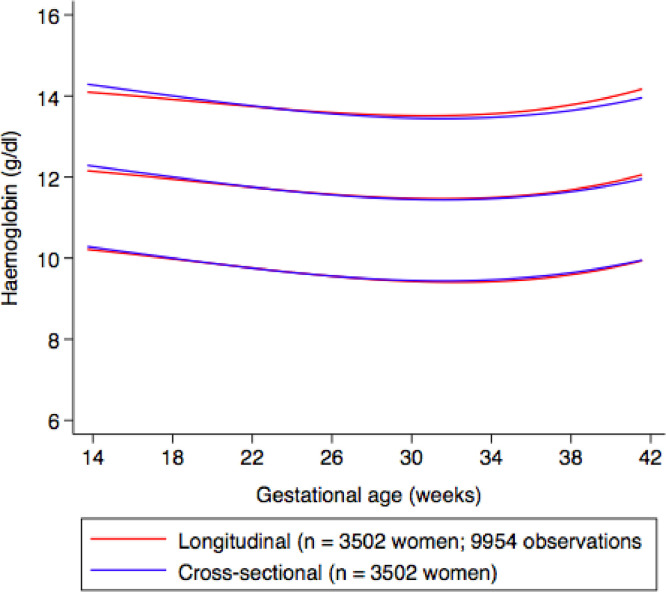
represents sensitivity analyses showing the smoothed, gestational age-specific, 3^rd^, 50^th^ and 97^th^ centiles for maternal haemoglobin from the total Fetal Growth Longitudinal Study using longitudinal data (*n* = 3502 women, 9954 observations) (red) compared to a cross-sectional random sample of haemoglobin data between 14^+0^ and 40^+0^ weeks’ gestation for each woman included in the study (*n* = 3502 observations) (blue). (For interpretation of the references to color in this figure legend, the reader is referred to the web version of this article.)

Sensitivity analyses excluding women who delivered preterm had no noticeable effect on the fitted 3^rd^, 50^th^ and 97^th^ centiles derived from the pooled sample (Fig. 6). There was a negligible effect on fitted centiles of excluding each site's data from the pooled analyses (Fig. 7). There were no differences in the fitted centiles using longitudinal and cross-sectional Hb data demonstrating negligible impact of alternative modelling of longitudinal data using multi-level models (Fig. 8).

The distribution of maternal Hb for those women reported to have been supplemented on iron (*n* = 2747), folic acid (*n* = 2972), calcium (*n* = 1788), either iron/calcium/folic acid (*n* = 3130), or any supplementation (*n* = 3213) were obtained from medical records and are shown in **Supplementary Figure 2**. No data on adherence is available.

1 **Thresholds for individual assessment and for population prevalence**

The equations for the mean and SD from the fractional polynomial models for maternal Hb according to exact gestational age in weeks are shown below:

**Table:** 

Mean SD	12.43805 – 0.0001386961*(GA^3^) + 0.0936269*((GA/10)^3^*log (GA/10)) 0.9853879 + 0.0033721*GA

All log are natural logarithms; GA = exact gestational age; SD = standard deviation

These equations allow for calculations by readers of any desired centiles according to gestational age in exact weeks. Any desired centiles can be calculated as mean ± *z* × SD where *z* = −1.88, −1.645, −1.28, 0, 1.28, 1.645 and 1.88 for the 3^rd,^ 5^th^, 10^th^, 50^th^, 90^th^, 95^th^ and 97^th^ centiles, respectively. The actual values for these centiles according to gestational age are presented in Table 3.

1 **Thresholds for individual assessment and for population prevalence where gestational age is unknown.**

For unknown gestational ages, the median maternal Hb centiles are as below:

**Table:** 

Median maternal Hb (in g/L)	3^rd^ centile	5^th^ centile	10^th^ centile	Median	90^th^ centile	95^th^ centile	97^th^ centile
	98	101	104	118	131	133	137

Finally, Table 4 summarises the proposed thresholds for individuals based on deviations from the normative median. For diagnostic purposes at the individual level, we propose three degrees of severity, with the lowest concentrations (which may suggest a diagnosis of anaemia) below the 3^rd^ centile.

**Table 4:** Thresholds for individuals according to deviations from the new normative trajectories for haemoglobin (Hb) in pregnancy.

INDIVIDUAL WOMEN (for clinical use)	
Gestational age-specific cut-off (normative centile)*	Probable diagnosis
<3^rd^ centile	Low Hb concentration
3^rd^ centile - 4.99^th^ centile	At high risk of low Hb concentration
5^th^ centile - 9.99^th^ centile	At moderate risk of low Hb concentration
≥10^th^ centile	Normal Hb

⁎ cut-offs derived from Fig. 4 and Table 3.

## 4 Discussion

The gestational age-specific centiles for maternal Hb presented here, are based on a population-based, prospective study throughout pregnancy of 3502 healthy, well-nourished women from eight countries, whose healthy babies were followed up to 2 years of age. The methods for selecting the sample of women adhered strictly to the WHO prescriptive approach used for the construction of the WHO Child Growth Standards [24,36], which supports the universal applicability of our findings.

Despite the many factors affecting Hb concentrations, we found remarkable similarities among populations compared with a large within-population variability, in common with other biomarkers of nutritional status [28,42,47]. We also observed a moderate nadir of maternal Hb between 31 and 32 weeks’ gestation as previously reported. The nadir by 31 weeks’ gestation resulted from a drop of 6.8 g/L at the 50^th^ centile compared to values at 14 weeks’ gestation, which is similar to the drop from <110 g/L to <105 g/L between the first and second trimesters in the current WHO and CDC guidelines for antenatal care [13,14].

To our knowledge, these are the first normative ranges of Hb values in pregnancy compatible with good, functional, maternal and perinatal outcomes, as well as neonatal and early childhood morbidity, growth and development up to 2 years of age. The 3^rd^ - 97^th^ centile range for maternal Hb is large across gestational ages (around 94 to 141 g/L) clearly demonstrating that healthy pregnant women have adaptive mechanisms to achieve adequate health outcomes within a wide range of Hb values. The resource should, therefore, act as a simple narrative to communicate to mothers the meaning of their routine blood test results.

This work also provides a system, at the individual level, for defining normal Hb distributions based on gestational age-specific cut-offs (<3^rd^ centile = low Hb concentration; 3rd - 4.99^th^ centile = high risk of low Hb concentration; 5^th^ - 9.99^th^ centile = moderate risk of low Hb concentration; ≥10^th^ centile = normal Hb concentration), an approach that is well accepted in biology and medicine. In addition, previous studies have suggested that high haemoglobin concentrations may be associated with increased pregnancy risks, including antepartum stillbirth and pre-eclampsia [48,49]. However, these findings are inconsistent, and in part this may be due to using a fixed cut-offs for defining haemoconcentration despite different gestational ages at the time of blood sampling. We believe that studies in this field can be facilitated by our work by allowing uniformity in gestational-age specific definitions of both low and high haemoglobin concentrations.

In short, the results presented here provide an international definition of low Hb concentrations in pregnancy, as well as a strong basis for constructing an international, pregnancy-specific, early warning score system to facilitate earlier recognition of deteriorating health in pregnant women. Our work in conjunction with other studies, that associate Hb concentrations with hypoxia-related outcomes [50], [51], [52], may provide data to create Hb concentration cut-offs for diagnosing anaemia at population level, although it will be necessary to establish which centiles reflect the different levels (mild, moderate or severe) of this important public health problem. The severity of anaemia determined by Hb concentration also needs to be determined on the basis of adverse outcomes, such as post-partum haemorrhage, the need for transfusion, or maternal or perinatal mortality.

Some thresholds recommended by WHO during pregnancy were proposed in 1958 and revised in 1968 after technical meetings with clinical and public health experts working with the evidence available at the time [11]. Those reports relied mostly on data aggregated from four European studies with very small sample sizes (UK, *n* = 45; Sweden, *n* = 50; Finland, *n* = 32; Finland, *n* = 267) [11,16–20]. Given the limited representativeness of those data and the scientific advances since made in understanding Hb biology [53], the ongoing WHO project on anaemia cut-offs will provide evidence-based guidance on Hb cut-offs for individuals and populations. The results from our work could help to build the body of evidence needed to help WHO to develop guidance for individual and public health programmes and policies.

To our knowledge, this is the first population-based study to collect prospective data from across the world using the prescriptive approach recommended by WHO for the construction of international standards for human growth and development [24]. A *prescriptive approach* shows how growth should occur, independent of time and place [54]. For human growth, this is usually based on selected populations considered to be of optimal health, for example, with adequate nutritional status and at low risk of abnormal growth. In contrast, the *descriptive approach* is usually based on an unselected population with minimal exclusion criteria such as known risk factors for optimal health [55,56]. We adopted a robust statistical methodology [39,56], as used in all the integrated studies of the INTERGROWTH-21^st^ Project [28,39], to pool our data and construct smoothed centiles that provide international gestational age-specific centiles for maternal Hb in healthy pregnancy.

By adopting a prescriptive approach for population selection and a pragmatic, yet highly standardised, approach to the design of the study, that mimics routine clinical practice in each of the eight diverse sites, we are confident that the international centiles are both statistically robust and representative of adult women of optimal health, nutrition, education, and socioeconomic status with uncomplicated pregnancies. Therefore, these Hb centiles represent normative values that public health measures should be targeted at achieving.

The proportion of total variance attributed to population differences among sites was only <13%, supporting the position that population-specific ranges for maternal Hb in pregnancy are not required. Furthermore, despite the great reduction in sample size when modelling Hb data for a single site separately, it was evident that there is less inter-site variation, and more so at the 3^rd^ centile. Our findings are further strengthened by the accuracy of gestational age estimation in the study cohort, all of whom had a confirmatory dating scan before 14 weeks’ gestation.

The study has some limitations. Firstly, we do not provide values for non-pregnant women. Secondly, we lacked pre-pregnancy and maternal Hb data <14 weeks’ gestation; however, a pre-pregnancy measure is rarely available in routine clinical practice so comparison with maternal Hb in early pregnancy has greater clinical applicability. Thirdly, because we took a pragmatic approach to the study design in relation to laboratory tests, we relied on collecting the results of available routine blood tests. The commercially available instruments for assessing Hb were not standardised across sites, however, all laboratories underwent standard laboratory quality assurance. Information on the use of preventive or therapeutic iron and folic acid supplements was collected from medical records. However, based on descriptive analyses, the risk of large systematic method differences was small. Fourthly, we do not provide values for women living in in high altitudes >1600 m. Lastly, the sample size, though large for a prospective study with repeated measures, the purposive selection of healthy women resulted in a relatively small sample to explore associations with maternal and neonatal outcomes such as preeclampsia.

The new Hb centiles allow comparisons across countries and regions using the same definitions and thresholds for potential interventions at population level, so as to harmonise efforts by WHO and other public health organisations to prevent and treat nutritional anaemias. Given the importance of evidence-informed interventions to improve maternal and child nutrition [57], we believe that national and international guidelines on antenatal care should take account of our findings. The fact that the centiles are gestational age-specific should also encourage policymakers to ensure that the gestational age of every pregnancy is estimated as accurately as possible, in line with the WHO 2016 guidelines [14].

Clearly, compared to the current recommendation [13,14], the derived haemoglobin distributions may have major policy implications because, once implemented, fewer pregnant women may be diagnosed with anaemia and the prevalence of low Hb levels will be substantially lower. This arises for two main reasons. Firstly, the current definition is derived from a statistical approach based on four very small studies, whereas the new derived haemoglobin distributions are compatible with the levels of functional health outcomes observed in the healthy populations we studied; secondly, we are presenting Hb trajectories according to gestational age rather than a single fixed cut-off across the whole of pregnancy (Fig. 4), which is an implausible biological concept. However, lowering the threshold for defining anaemia towards the end of pregnancy at individual level might have implications for management; for instance, recognising lower thresholds may allow more targeted treatment for anemia, and may alter management in those women who then have a post-partum haemorrhage.

Finally, considering the indisputable need for pregnancy-specific early warning scores that incorporate maternal Hb changes [3,22] our findings could be used to facilitate earlier recognition and treatment of the unwell pregnant woman and thereby reduce both maternal morbidity and mortality rates worldwide.

Future research that focuses on obtaining accurate data on plasma volume expansion (using non-invasive methods) during healthy pregnancies would be ideal as this parameter has an impact on many biomarkers in pregnancy. Based on these derived gestation-specific centiles, a study that evaluates the statistically derived cut-offs including upper limit cut-offs and the perinatal risk-associated cut-off levels using large datasets with sufficient severe morbidity and mortality events would help to establish and validate the derived thresholds and would add support to more evidence-based protocols for the care of pregnant women.

To our knowledge, this is the first attempt to produce maternal Hb thresholds based on populations selected using the WHO prescriptive approach for the construction of international standards. We present international, gestational age-specific centiles for Hb in pregnancy, which are compatible with good, functional, maternal and perinatal outcomes, as well as neonatal and early childhood morbidity, growth and development up to 2 years of age.

## Declaration of Competing Interest

ATP reports personal fees from BJOG, grants from Bill and Melinda Gates Foundation, grants from European Research Council, grants from National Institute of Health Research, grants from Grand Challenges Research Fund, outside the submitted work. JPPR and MNGC are full time staff members of the WHO. The authors alone are responsible for the views expressed in this publication and they do not necessarily represent the official position, decisions, policy or views of the World Health Organization. The WHO receives partial financial support from the Bill & Melinda Gates Foundation, and the US Centers for Disease Control and Prevention to support its work in the area of nutrition. Donors do not fund specific guidelines and do not participate in any decision related to the guideline development process. All other authors declare no competing interests.

## Appendix Supplementary materials

Image, application 1
