# Supplementary material for: International values for haemoglobin distributions in healthy pregnant women
Source: eClinicalMedicine. 2020 Dec 2;29-30:100660. doi: 10.1016/j.eclinm.2020.100660 (PMC7788439; doi:10.1016/j.eclinm.2020.100660)
Supplement: Supplementary file 1 [file mmc1.docx]

**Supplementary Table 1. Anthropometric measures at 2 years of age in children who contributed data to the international estimated haemoglobin values proposing new thresholds for apparently healthy women in pregnancy compared with the World Health Organization Child Growth Standards^*^**

|  |  | **Present analysis: INTERGROWTH-21^st^ cohort** | | **INTERGROWTH-21^st^ cohort expressed according to the WHO Child Growth Standards** | |
| --- | --- | --- | --- | --- | --- |
| **Variable** | **N** | **Mean^+^ ± SD**^‡^ | **Median (interquartile range)** | **Mean z-score ± SD** | **Median centile** |
| **Weight (kg)** | 2601 | 12.3 (1.7) | 12.1 (11.1, 13.3) | 0.2 (1.1) | 56 |
| **Length (cm)** | 2584 | 87.4 (3.6) | 87.3 (85.0, 89.8) | 0.1 (1.1) | 55 |
| **Head circumference (cm)** | 2602 | 47.7 (1.6) | 47.7 (46.6, 48.8) | -0.1 (1.1) | 48 |

*Age and gender specific z-scores and percentiles compared with the WHO Child Growth Standards; ^+^ Mean values were estimated from raw data; ^‡^ SD = Standard Deviation.

**Supplementary Table 2. Morbidity at 1 and 2 years of age of children who contributed data to the international estimated haemoglobin values proposing new thresholds for apparently healthy women in pregnancy**

| **Medical condition** | **1 year of age** | **2 years of age** |
| --- | --- | --- |
|  | N = 2320 (%) | N = 2650 (%) |
| Hospitalised at least once | 278 (11.4) | 237 (8.9) |
| Total no. of days hospitalised * | 3 (1, 6) | 2 (1, 4) |
| Any prescription made by a health care professional | 1452 (59.7) | 1669 (63.0) |
| Antibiotics (≥ 3 regimens) | 270 (11.1) | 427 (16.1) |
| Up-to-date with local vaccination policies | 2225 (91.5) | 2542 (96.0) |
| Otitis media/pneumonia/bronchiolitis | 208 (8.6) | 267 (10.1) |
| Parasitosis/diarrhoea/vomiting | 123 (5.1) | 125 (4.7) |
| Seizures/cerebral palsy/neurological disorders | 6 (0.3) | 6 (0.2) |
| Exanthema/skin disease | 360 (14.8) | 332 (12.5) |
| UTI^+^ /pyelonephritis | 5 (0.2) | 9 (0.3) |
| Fever ≥ 3 days (≥ 3 episodes) | 255 (10.5) | 277 (10.5) |
| Malaria | 5 (0.2) | 6 (0.2) |
| Meningitis | 8 (0.3) | 1 (<0.1) |
| Other infections that required antibiotics | 59 (2.4) | 68 (2.6) |
| Hearing problems | 4 (0.2) | 2 (0.1) |
| Asthma | 15 (0.6) | 30 (1.1) |
| Cardiovascular problems | 6 (0.3) | 6 (0.2) |
| Blindness | 5 (0.2) | 3 (0.1) |
| Gastro-oesophageal reflux | 72 (3.0) | 8 (0.3) |
| Any haemolytic condition | 14 (0.6) | 21 (0.8) |
| Any malignancy | 2 (0.1) | 6 (0.2) |
| Cow’s milk protein allergy | NA^‡^ | 17 (0.6) |
| Food allergies | NA^‡^ | 44 (1.7) |
| Injury trauma | 41 (1.7) | 120 (4.5) |
| Any condition that required surgery | 22 (0.9) | 28 (1.1) |

*Data are given as median (interquartile range); ^+^ UTI = Urinary tract infection; ^‡^ NA, not applicable (data were not collected at the 1-year follow-up visit)

**Supplementary Figure 1. Median age of achievement (3^rd^ and 97^th^ centiles) of four gross motor development milestones. Data are for children who contributed data to the proposed international estimated haemoglobin values for apparently healthy women in pregnancy. For comparison, the 3^rd^ and 97^th^ centiles of the World Health Organization windows of achievement for the same milestones (36) are presented in *grey* (with the median shown as a *vertical line*).**

**Supplementary Figure 2: represents the distribution of maternal haemoglobin (in g/L) obtained from medical records of 3502 women participating in the Fetal Growth Longitudinal Study who reported consuming supplements containing iron (n=2747), folic (n=2972), calcium (n=1788) (either iron/calcium/folic acid, n=3130), or any other supplements (n=3213) during pregnancy**
